# Glycemic Control during Pregnancy—A Predictor of Vitamin C Status at Labor in Type 1 Diabetic Women?

**DOI:** 10.3390/antiox8060153

**Published:** 2019-05-31

**Authors:** Bente Juhl, Finn F. Lauszus, Jens Lykkesfeldt

**Affiliations:** 1 Medical Department, Aarhus University Hospital, 8200 Aarhus, Denmark; 2 Gynecology & Obstetrics Department, Herning Hospital, 7400 Herning, Denmark; Finn.Friis.Lauszus@vest.rm.dk; 3 Faculty of Health and Medical Sciences, University of Copenhagen, 2200 København N, Denmark; jopl@sund.ku.dk

**Keywords:** type 1 diabetes mellitus, HbA1c, pregnancy, vitamin C, cohort study

## Abstract

Several experimental studies have suggested that vitamin C (vitC) deficiency during pregnancy may be detrimental to fetal development, and observational studies have shown that vitC status is lower during pregnancy and in people with diabetes. A cross-sectional study in pregnant type 1 diabetic women found that poor maternal vitC status was a significant predictor for obstetric complications of pregnancy when measured within four weeks before labor. The plasma vitC concentration was significantly negatively correlated to HbA1c, the biomarker of glycemic control well-known to be associated with the outcome of the diabetic pregnancy. Here, we evaluated HbA1c during pregnancy in relation to the measured vitC levels in late pregnancy based on data from 46 women from the same cohort. Regression analysis showed that HbA1c of first trimester, the combined mean HbA1c of first and second trimester, mean HbA1c of the whole pregnancy (first, second and third trimester combined), and HbA1c of third trimester alone were all associated with vitC in late pregnancy (*p* = 0.03, *n* = 45; *p* = 0.034, *n* = 43; *p* = 0.017, *n* = 42; and *p* = 0.008, *n* = 46, respectively). In third trimester, when adjusted for creatinine clearance, the association between vitC and HbA1c persisted (*p* = 0.029). Women in third trimester with HbA1c above 7.0% had an increased risk of having poor vitC status compared to women with HbA1c below this level (11 out of 21 vs. 2 out of 25 women, *p* < 0.001). The results suggest that high HbA1c is associated with poor maternal vitC status and potentially inadequate supply of vitC for the neonate. HbA1c may thus be a relevant substitute biomarker for identifying pregnant women who might benefit from vitC supplementation.

## 1. Introduction

In type 1 diabetes mellitus (T1DM), vitamin C (vitC) levels are significantly lower than in non-diabetic subjects [1,2]. As recently reported by us, this seems also to be the case in the diabetic pregnancy [3]. We found even lower plasma vitC concentrations than in non-pregnant T1DM patients [4], probably owing the well-known preferential placental transport of vitC and rapid fetal growth that tends to lower the maternal vitC status during pregnancy per se [5]. Thus, poor vitC status defined as a plasma vitC concentration < 23 µmol/L [6] was found in 51% of the diabetic women at some stage during pregnancy [3]. Moreover, we also found that vitC status in late pregnancy was significantly negatively correlated to the level of glycemic control as measured by HbA1c, a relationship that has not previously been described in the literature [7]. 

Data from the DAPIT study have suggested that women with T1DM who develop pre-eclampsia have significantly higher HbA1c before and during pregnancy compared with women who do not develop pre-eclampsia [8]. In line with this, the risk of preterm delivery, stillbirth, and a composite adverse outcome increase progressively with increasing HbA1c level above 6.5%, indicating the potential clinical use of HbA1c measurements throughout pregnancy in predicting adverse outcome [9]. In fact, HbA1c above 5.9% is a marker of hyperglycemia in pregnancy and was found to be associated with adverse pregnancy outcome, thus having major implications for mother and child [10,11,12]. However, a possible relationship to vitC metabolism has only scarcely been addressed. 

Here, we report an evaluation of HbA1c during pregnancy in relation to the vitC status in late pregnancy in a cohort of pregnant T1DM women, where poor vitC in late pregnancy was previously found to be a significant predictor of complications of pregnancy [7].

## 2. Materials and Methods

From June 1992 to August 1994, 76 women with T1DM attending the Department of Obstetrics, Aarhus University Hospital, were included in a study on vitC during pregnancy and compared to controls, as described previously [3]. The inclusion criteria were pregestational T1DM, age >18 years, no other systemic disease than diabetes, and singleton pregnancy.

The study was part of an evaluation of morbidity in diabetic pregnancy with respect to nephropathy and retinopathy and was approved by the local Ethical Committee (jr.nr.1992/2523, 1998/4147, and 2026-99). It was performed in concordance with the Helsinki II declaration, and all women had given their informed consent. The women were recommended to take multivitamins and folic acid in early pregnancy, in accordance with common practice at Aarhus University Hospital at the time of the study (later extended to the entire country), and were not specifically informed about the focus on vitC levels during pregnancy in the present study to avoid potential bias. Dietary data were not recorded. The women were informed that we wanted to evaluate the relevance of various biomarkers during pregnancy in T1DM. The study was approved by the local Ethical Committee (jr.nr. 1992/2328).

In the present study, we focused on glycemic status as a marker of hypovitaminosis C. Thus, the predictive value of the mean HbA1c in third, the mean of HbA1c in first, the mean of the sum of HbA1c in first and second, and the mean HbA1c of the whole pregnancy (*n* = 46, 45, 43 and 42, respectively) were evaluated in relation to the vitC status late in pregnancy (within four weeks of delivery) based on the data originally obtained. We have previously used data from the same cohort of women with T1DM comparing vitC levels with those of healthy controls [3] and relation to labor data and obstetrical outcome of pregnancy [7]. If more than one sample of HbA1c per trimester was measured, the sample mean was used in the data analysis. Sufficient vitC data on all measured time points were present in 46 of the initial 76 women.

HbA1c was measured by a commercial kit (BioRad, Richmond, CA, USA) according to the manufacturer’s instructions. Blood samples for plasma vitC analysis were stabilized in sodium EDTA-anticoagulated vacutainer tubes containing dithiothreitol. Tubes were centrifuged, and plasma was removed and deproteinized by addition of 6% perchloric acid. Samples were kept at −80 °C before analysis and assayed by high-performance liquid chromatography [13]. The women were included within one year, and all samples were analyzed in one batch in 1994. Details of the analytical performance were published previously [3]. No other vitamins or minerals were analyzed.

Clinical data and diabetic characteristics that were found significantly associated with vitC in late pregnancy were subsequently included in the multiple regression analysis regarding vitC in late pregnancy as the dependent variable. We carried out a predefined HbA1c-subgroup analysis using the threshold of HbA1c at 7.0% (= 53 mmol/mol), a level found useful in predicting complications of pregnancy in a cohort of 1094 type 1 diabetic pregnancies [14]. The analysis used first the mean of HbA1c of the whole pregnancy (first, second and third trimester combined), the mean HbA1c of first and second combined, and lastly the mean of first and third for evaluating the utility of HbA1c in late pregnancy in predicting poor vitC status (defined as <23 µmol/L) measured within four weeks before labor.

Statistics were performed with SigmaPlot 12, Systat software. Values are given as mean (SD) if not otherwise stated. Qualitative data were analyzed by Fisher’s exact test. Linear regression and multiple linear regressions were used as predictive analysis of quantitative data. A two-sided *p* < 0.05 was chosen as level of significance. 

## 3. Results

Clinical data from the pregnant women with T1DM are shown in Table 1. Creatinine clearance at the end of pregnancy correlated positively with vitC status in late pregnancy (*r*² = 0.19, *p* = 0.018, *n* = 30) and was consequently included in the regression analysis. 

Women with HbA1c ≤ 7.0% in late pregnancy had a significantly lower relative risk of poor vitC status compared to women with HbA1c above this level (2 out of 25 vs 11 out of 21 women, *p* < 0.001). Thus, the threshold for HbA1c of >7.0% within four weeks before labor was able to identify diabetic women at significantly increased risk of hypovitaminosis C (from 8% to 52% in late pregnancy), while the threshold of 7.0% in subgrouping the mean of HbA1c of the entire pregnancy was non-significant in identifying women characterized by hypovitaminosis C. 

**Figure 1:**
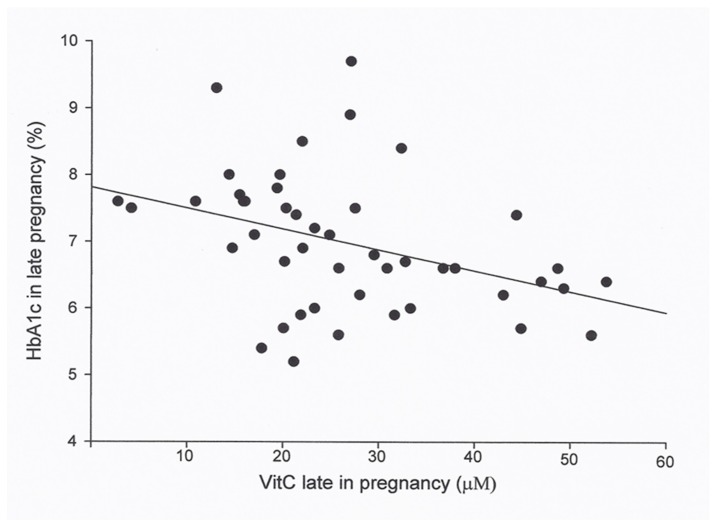
Plasma vitamin C (vitC) in 46 women with type 1 diabetes in late pregnancy in relation to the HbA1c in late pregnancy. Regression analysis showed that the means of HbA1c in late pregnancy was associated with vitC in late pregnancy (*r*² = 0.15, *p* = 0.008, *n* = 46), and the association persisted when adjusted for creatinine clearance third trimester (*r*² = 0.31, *p* = 0.029, power of test >80%).

As outlined in Table 2 and Figure 1, regression analysis showed a significant correlation between HbA1c during pregnancy and plasma vitC in late pregnancy in 46 women with type 1 diabetes. The mean sum of HbA1c of the whole pregnancy was associated with the mean vitC of the whole pregnancy (*p* = 0.027; *n* = 36) but not when adjusted for the mean creatinine clearance of pregnancy (*p* = 0.067, power of test 61%).

## 4. Discussion

In the present study, we found that HbA1c in first trimester and later during pregnancy was negatively correlated to the maternal vitC level at delivery, and that HbA1c level of 7.0% offered a threshold, above which women within four weeks before labor had significantly increased risk of hypovitaminosis C, a prevalence more than six-fold higher than for those with an HbA1c below 7.0%. Thus, HbA1c may constitute a robust marker of hypovitaminosis C in late pregnancy. 

The relationship between hyperglycemia and increased risk of detrimental obstetrical and fetal outcome as well as later diabetic complications is well-established and is generally accepted as causative [15,16,17]. Consequently, the relationship to plasma vitC status may just express a para-phenomenon and no causality. However, in experimental studies in guinea pigs that, like humans, depend on an adequate supply of vitC through their diet, the offspring of vitC deficient dams showed abnormalities similar to those observed in diabetic animals, and these abnormalities were ameliorated by vitC supplementation [18,19,20,21]. A study in pregnant sodium-dependent vitC transporter 1 (SVCT1) knockout mice has shown a fetal loss of about 50% at low vitC but at concentrations above scurvy level, a loss that was found to be prevented by increasing dietary vitC levels [22]. 

The mechanisms by which hyperglycemia per se cause diabetic late complications and complications of the diabetic pregnancy are not resolved. However, one possible mechanism includes increased urinary ascorbic acid clearance due to hyperglycemia-impaired tubular reabsorption of filtered vitC, as found in people with diabetes, even though the level of vitC among these patients is often well below the renal threshold promoting excretion of vitC through the urine in healthy individuals [23,24,25,26]. Another link between poor vitC status and diabetic complications could be hyperglycemia-induced oxidative stress [27,28], a stress condition that increases the turnover of vitC, as also seen in smokers and in other chronic inflammatory conditions [29,30,31,32,33,34]. 

Indeed, the intake of vitC among smokers needed to maintain a steady state condition comparable to that of non-smokers is considerably higher [35,36], and the same situation may apply to the diabetic state, a need that is probably further increased in the diabetic pregnancy owing to the considerable transport of vitC to the fetus. Interestingly, both maternal smoking and autoimmune chronic inflammatory diseases are conditions that are characterized by increased risk of complications of pregnancy and adverse neonatal outcomes [37,38,39]. The complications resemble those observed in our previous examination of hypovitaminosis C and diabetes in the human pregnancy, respectively [7]. In the present study, we were not able to ascertain the number of cigarettes or if maternal smoking continued throughout pregnancy. Consequently, we cannot address the effect of smoking on vitC status.

Limitations of the present study include the small number of participants and that dietary data and supplement use were not recorded. The included T1DM patients with diabetic complications, i.e., retinopathy and micro- and macroalbuminuria, may influence the observed relations between glycemic control and vitC status in late pregnancy. We found, consistent with that notion, a positive correlation of creatinine clearance with vitC in late pregnancy. However, creatinine clearance per se did not predict complications of pregnancy. Finally, the samples for vitC were taken in a non-fasting state to avoid hypoglycemic episodes, which may increase the variance of the vitC measurements and tend to conceal associations.

## 5. Conclusions

In conclusion, the results from a cohort of pregnant women with T1DM suggest that a high HbA1c during pregnancy is associated with poor maternal vitC status in late pregnancy. Further investigations are needed to describe the possible clinical significance of glycemic control in relation to poor vitC status in diabetic pregnancy and if vitC supplementation would be of benefit in these women.

## Figures and Tables

**Table 1:** Clinical data and characteristics of the study population. Values are reported as mean (SD) for normally distributed quantitative data or as frequencies for qualitative data.

Variable	*n*	Value
Age (yr)	46	28.2 (3.6)
Diabetes duration (yr)	46	14.1 (8.9)
Parity	46	1.8 (0.7)
Systolic blood pressure at entry (mmHg)	28	120.0 (9.9)
Diastolic blood pressure at entry (mmHg)	28	71.2 (6.9)
Retinopathy (n = No/Simplex/Proliferative)	46	23/17/6
BMI at delivery (kg/m²)	31	28.6 (4.3)
Normo-/Micro-/Macro-albuminuria (n/n/n)	46	37/8/1
HbA1c in first trimester (%) ¹	45	7.6 (1.2)
HbA1c in second trimester (%) ¹	43	7.0 (0.9)
HbA1c in late pregnancy (%) ¹	46	7.0 (1.0)
HbA1c in first and second trimester combined (%)	43	7.3(1.1)
HbA1c mean of the whole pregnancy (%)	42	7.2(0.9)
VitC in late pregnancy (µmol/L)	46	30.1(13.8)
VitC mean of pregnancy (µmol/L)	37	34.8(14.0)
Complications of pregnancy (n = yes/no) ²	46	19/27
Creatinine clearance at entry (ml/min)	35	122.1 (22.1)
Creatinine clearance at delivery (ml/min)	31	99.2 (33.2)
Smoker (n = yes/no/unknown)	46	15/30/1

¹ Comparison of the means of HbA1c in first, second and in late pregnancy showed a significant decrease from first to second (SEM: 0.18 versus 0.16) and to HbA1c in late pregnancy (SEM: 0.18 versus 0.15) (*p* < 0.01); ² for further details, see [7].

**Table 2:** Results of regression analysis of HbA1c during pregnancy in relation to the dependent variable vitC in late pregnancy in our study cohort.

Independent Variable	*r*²	*n*	*p* Value
HbA1c in first trimester (%)	0.11	45	0.03
HbA1c in first and second trimester (%)	0.14	43	0.034
HbA1c of the whole pregnancy (%)	0.18	42	0.017(0.055) *²
HbA1c in late pregnancy (%)	0.15	46	<0.01(0.029) *¹

* When adjusted for creatinine clearance in third trimester, the association between HbA1c and vitC in late pregnancy persisted; *¹ (*r*² = 0.31, *p* = 0.029, power of test >80%; see Figure 1), while calculated for the entire pregnancy, the correlation did not reach significance; *² (*p* = 0.055, power of test 67%).
